# Case Report: Invasive pulmonary aspergillosis caused by *Aspergillus lentulus* in a boy with chronic granulomatous disease

**DOI:** 10.3389/fmed.2026.1813957

**Published:** 2026-06-02

**Authors:** Shan Tan, Qiong Liao, Yang Wen, Yu Zhu

**Affiliations:** 1 Department of Pediatric Infectious Diseases, West China Second University Hospital, Chengdu, China; 2 Key Laboratory of Birth Defects and Related Diseases of Women and Children (Sichuan University), Ministry of Education, Chengdu, China; 3 NHC Key Laboratory of Chronobiology (Sichuan University), Chengdu, China

**Keywords:** anti-fungal therapy, *Aspergillus lentulus*, boy, chronic granulomatous disease, invasive pulmonary aspergillosis

## Abstract

*Aspergillus lentulus* is a slow-growing and drug-resistant fungus, which has been primarily reported in adults, usually immunocompromised ones, suffering from invasive pulmonary aspergillosis (IPA). This condition is rare in children. Here, we report a case of invasive pulmonary aspergillosis due to *Aspergillus lentulus* in a boy with no history of recurrent infections who presented with a prolonged fever of unknown origin. Based on chest CT scan findings showing typical halo signs, a fungal infection was strongly suspected. Empirical antifungal therapy was initiated at early admission but failed to resolve the persistent fever in this case. The causative pathogen was confirmed by blood metagenomic next-generation sequencing (mNGS). Subsequent genetic analysis identified a pathogenic mutation in the X-linked CYBB gene, confirming chronic granulomatous disease (CGD). Eventually, following a combination therapy of voriconazole and micafungin, the boy became afebrile and was discharged, pending hematopoietic stem cell transplantation (HSCT). To our knowledge, no previous cases of *Aspergillus lentulus* infection in children with CGD have been reported in the literature. This case underscores the critical importance of identifying the causative microorganism. It also highlights the value of emerging detection methods, such as mNGS. At present, there is no consensus for the optimal antifungal regimen against pediatric *Aspergillus lentulus* infections. Clinical improvement was achieved in this patient following combination therapy with voriconazole and micafungin, offering a practical therapeutic reference for managing this refractory fungal infection.

## Introduction

*Aspergillus lentulus* is a slow-growing, drug-resistant fungal species that is relatively rare. Invasive aspergillosis is caused by *A. lentulus*, and, in the majority of cases, it occurs in immunocompromised adults (1, 3, 5). This condition is relatively uncommon in the pediatric population and is known for having high minimum inhibitory concentrations (MICs) to all triazoles and amphotericin B (1–3), which poses significant challenges to clinical treatment. Immunocompromised hosts, including those with chronic granulomatous disease (CGD), chemotherapy recipients, and organ transplant recipients, are at heightened risk of developing invasive aspergillosis secondary to impaired immune defense mechanisms. As a primary immunodeficiency disorder, CGD is characterized by defective neutrophil bactericidal function, a hallmark that markedly enhances susceptibility to invasive fungal infections. Given the relative scarcity of *Aspergillus lentulus* infections in pediatric patients and the lack of consensus on the optimal antifungal regimen for these cases, this case report underscores the critical importance of identifying the causative microorganism and highlights the value of emerging detection methods, such as metagenomic next-generation sequencing (mNGS). Clinical improvement was achieved in this patient following a combination therapy of voriconazole and micafungin, offering a practical therapeutic reference for managing this refractory fungal infection.

## Case report

**FIGURE 1:**
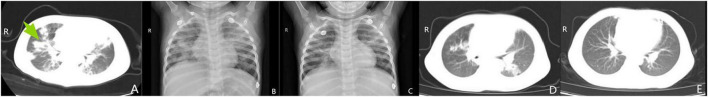
Chest computed tomography (CT) scan and chest X-ray findings **(A)**. The chest CT scan taken at admission showed multiple masses and nodules with halo signs (arrows), suggestive of an invasive fungal infection in both lungs, along with enlarged bilateral hilar and mediastinal lymph nodes. The findings from the chest X-ray worsened when vancomycin was combined with voriconazole **(B)**. The lung images gradually improved when micafungin was added to the voriconazole treatment **(C,D)**. The boy was finally discharged with a prescription for oral voriconazole and sulfonamide. In our last follow-up, the CT scan showed evident improvement **(E)**.

A 2-year, 4-month-old boy with a 44-day history of fever was admitted to the Pediatric Infectious Unit at West China Second University Hospital, a tertiary teaching hospital. He was previously healthy with no history of recurrent infections. He had chills and rigor before the fever, but no respiratory symptoms or signs when admitted. His vital signs were as follows: body temperature 36.5°C, pulse rate 130 beats/min, respiratory rate 32 breaths/min, body weight 10.6 kg, and height 85 cm. Physical examination revealed mild hepatomegaly with no other positive findings. Routine testing showed an elevated white blood cell (WBC) count (17.4 mm^3^) and C-reactive protein (CRP) level (82 mg/L), and a chest computed tomography (CT) scan showed multiple masses and nodules with halo signs in both lungs and enlarged bilateral hilar and mediastinal lymph nodes (Figure 1A). Screenings for Mycobacterium tuberculosis, *Mycoplasma pneumoniae*, and other bacteria and viruses in the sputum were negative. Serum (1,3)-β-D-glucan, galactomannan, and cryptococcal antigen lateral flow assay testing showed negative results.

The patient was treated with intravenous ceftriaxone but did not respond to the therapy. Although he had a previously healthy history and no direct experimental evidence of fungal infection, a diagnosis of invasive pulmonary aspergillosis was suspected, based on the CT findings and clinical symptoms. On hospital day 5, the patient was treated with the antifungal voriconazole (8 mg/kg per 12 h). On hospital day 6, the patient underwent bronchoscopy, with blood and bronchoalveolar lavage fluid (BALF) obtained simultaneously for metagenomic next-generation sequencing (mNGS; Hugobiotech, China) analysis. On hospital day 15, blood mNGS identified *Aspergillus lentulus* with three specific sequence reads, while BALF mNGS detected *Streptococcus pneumoniae*, a common respiratory pathogen. Low-abundance CMV was detected in the non-sterile BALF sample. Its viral load was well below the clinical diagnostic threshold, and the patient showed no compatible clinical symptoms or corresponding laboratory abnormalities suggestive of CMV infection. CMV was therefore interpreted as a commensal virus of no clinical significance, and no targeted antiviral therapy was given. An invasive fungal infection caused by this uncommon pathogen in an otherwise healthy child raised suspicion of an underlying primary immunodeficiency and prompted subsequent genetic analysis. His peak temperature dropped to approximately 38.6°C, and the frequency of fever was reduced to once a day. Although we combined vancomycin with voriconazole, the patient developed a cough, rales, and exacerbations in his lungs, as demonstrated by an X-ray (Figure 1B). Based on previous reports (1, 4, 5), on hospital day 19, we added micafungin (3 mg/kg once daily) to the voriconazole regimen. Subsequently, the patient’s cough and lung signs improved. Chest radiography after 9 days of combination treatment showed improvement (Figure 1C), and a chest CT scan performed a further 6 days later revealed additional radiological resolution (Figure 1D). After a total of 18 days of combination therapy, the antifungal regimen was switched to voriconazole monotherapy.

**FIGURE 2:**
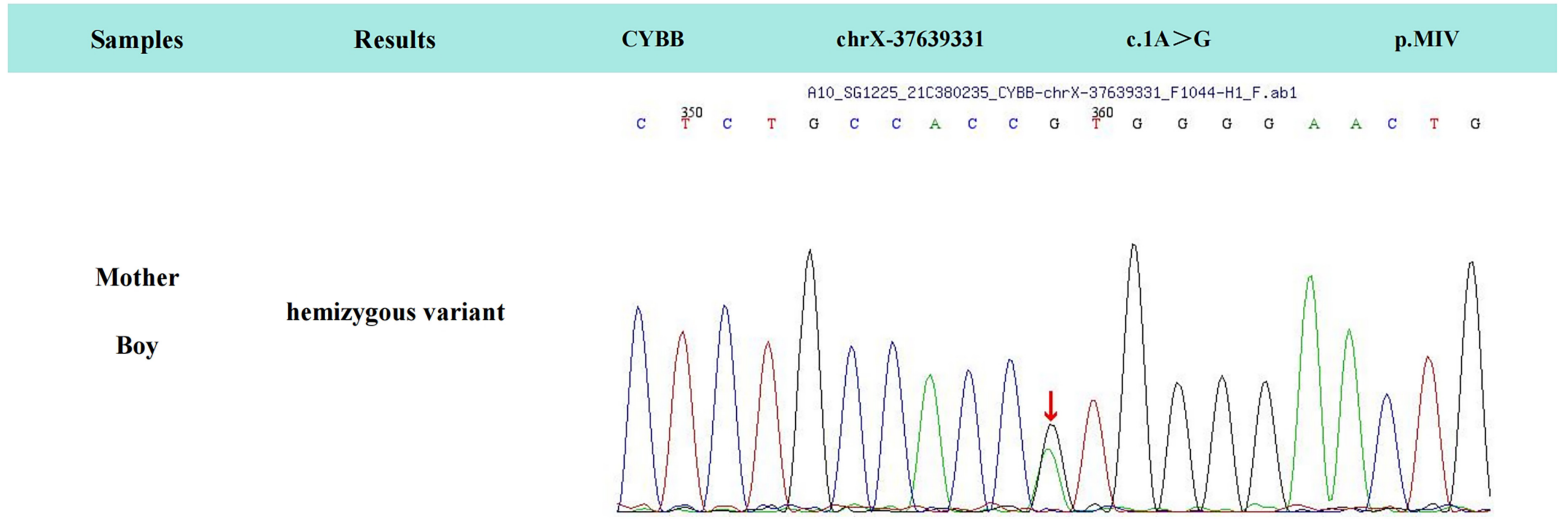
Sanger sequencing analysis of the CYBB gene in the patient and his mother. The patient inherited a pathogenic hemizygous variant (c.1A > G) in the X-linked CYBB gene from his mother, confirming the diagnosis of chronic granulomatous disease (CGD).

Genetic analysis using a primary immunodeficiency gene panel (MyGenostics, China) identified a pathogenic mutation in the X-linked CYBB gene in this patient (Figure 2), confirming the diagnosis of CGD (6). On hospital day 32, we gave the child oral voriconazole and sulfonamide, along with intravenous piperacillin-tazobactam. His temperature was completely normal for 1 week, and he was discharged with oral voriconazole and sulfonamide after 44 days of hospitalization. At our 5-month follow-up, his temperature was normal, and the CT scan showed significant improvement (Figure 1E). Currently, the boy is awaiting hematopoietic stem cell transplantation (HSCT).

### mNGS methodology

Peripheral blood and bronchoalveolar lavage fluid (BALF) samples were collected with sterile tubes and sent to Hugobiotech Co., Ltd. (Beijing, China) for mNGS detection. DNA was extracted from the samples using a QIAamp DNA Micro Kit (QIAGEN, Hilden, Germany) following the manufacturer’s instructions. DNA libraries were constructed with the QIAseq™ Ultralow Input Library Kit for Illumina (QIAGEN, Hilden, Germany), following the manufacturer’s instructions. The Qubit fluorometer (Thermo Fisher Scientific, MA, USA) and an Agilent 2100 Bioanalyzer (Agilent Technologies, Palo Alto, USA) were utilized to assess the quality and quantity of each library. Only those libraries that met the quality standards were sequenced on the Nextseq 550 platform (Illumina, San Diego, USA), with a target of approximately 20 million 75 bp single-end reads per library.

For bioinformatics analysis, the raw data were filtered to remove adapter sequences, short reads (<35 bp), low-quality reads (Q score <30), and low-complexity reads. Human reads were excluded by mapping them to the human reference genome (hg38). The remaining reads were aligned to Microbial Genome Databases using the Burrows-Wheeler Aligner.

Negative controls (NTC, sterile deionized water) and positive controls (known quantities of synthetic fragments) were included in each batch of experiments and underwent the same wet lab and bioinformatics procedures. For detected bacteria (excluding Mycobacterium tuberculosis), fungi (excluding Cryptococcus), and parasites, a positive result was considered if the coverage ranked in the top 10 among similar species/genera and was absent in the NTC or if the RPM ratio between the sample and NTC (RPMsample/RPM NTC) was greater than 10 when the RPM NTC was not zero. For viruses, Mycobacterium tuberculosis, and Cryptococcus, a positive mNGS result was indicated by the detection of at least one species-specific read that was absent in the NTC or by an RPM sample/RPM NTC ratio greater than 5 when the RPMNTC was not zero.

## Discussion

To our knowledge, this case represents the first reported case of an invasive pulmonary infection caused by *Aspergillus lentulus* in a child with CGD. *Aspergillus lentulus* is a slow-growing, drug-resistant species that is relatively rare and has previously only been reported in immunocompromised adults (1, 4, 7). It is known to have high minimum inhibitory concentrations (MICs) to all triazoles and amphotericin B (1–3). Consistent with reports by Balajee et al. and Nematollahi et al., our isolate was suspected to be resistant, which guided our decision to use combination therapy (2, 3). Our patient was a young child with no specific past medical history. Therefore, what we learned from this patient is never to ignore the diagnostic clues of invasive aspergillosis suggested by the imaging features.

*Aspergillus lentulus* is morphologically highly similar to *Aspergillus fumigatus*, which often leads to misidentification by conventional culture and phenotypic methods (2, 5). Diagnostic challenges were prominent in this case, as conventional mycological examinations, including the GM test and fungal culture, yielded negative results. In this case, mNGS played a critical role in the accurate identification of *Aspergillus lentulus*, thus allowing for timely and targeted antifungal management (8). mNGS is becoming more and more available for the diagnosis of difficult infection cases clinically.

The disease progressed slowly while the boy exhibited signs of infection. However, he only had a partial response to voriconazole monotherapy for aspergillosis. Given the triazole resistance inherent to *Aspergillus lentulus* and the suboptimal response to single-agent voriconazole, a combination therapy of voriconazole and micafungin was administered. Based on the above features, the pathogenic organism was confirmed, and the therapeutic strategy proved effective. This case indicates that combination therapy with voriconazole and micafungin could be an effective therapeutic strategy for invasive *Aspergillus lentulus* infections in pediatric patients.

For young children, even if they have been healthy in the past, primary immunodeficiency disease should be considered when they suffer from unusually serious infections. One example in this case is CGD (9, 10). Hematopoietic stem cell transplantation (HSCT) may be a feasible treatment for patients with CGD and invasive pulmonary aspergillosis (11).

This case underscores the critical importance of identifying the causative microorganism and highlights the value of emerging detection methods, such as mNGS. At present, there is no consensus on the optimal antifungal treatment regimens for *Aspergillus lentulus* infections in children. Clinical improvement was achieved in this patient following combination therapy with voriconazole and micafungin, offering a practical therapeutic reference for managing this refractory fungal infection.

## Limitations

As a single case report, the findings are not generalizable. The lack of *in vitro* susceptibility data for the isolate is also a limitation. Furthermore, the long-term outcome of the combination therapy requires further follow-up.

## Conclusion

In conclusion, this first reported pediatric case of an *Aspergillus lentulus* infection in a CGD patient highlights (1) the importance of considering fungal infections based on imaging, even in healthy-appearing children; (2) the utility of NGS in identifying drug-resistant pathogens; and (3) the potential efficacy of a voriconazole-micafungin combination therapy. This case also reinforces the need to investigate for primary immunodeficiencies, such as CGD, in children with severe, opportunistic infections.

## Data Availability

The original contributions presented in this study are included in this article/supplementary material, further inquiries can be directed to the corresponding author.
